# Prognostic value of the GRANT score and development of a nomogram in papillary renal cell carcinoma: a SEER-based study with external validation in a Chinese cohort

**DOI:** 10.3389/fonc.2025.1659055

**Published:** 2025-11-05

**Authors:** Tongpeng Liu, Wei Chen, Yu Yao, Yang Hu, Lijiang Sun, Guiming Zhang

**Affiliations:** Department of Urology, The Affiliated Hospital of Qingdao University, Qingdao, China

**Keywords:** papillary renal cell carcinoma, GRANT score, nomogram, prognosis, SEER database, external validation, cancer-specific survival

## Abstract

**Background:**

Papillary renal cell carcinoma (pRCC) exhibits significant heterogeneity, and robust prognostic tools specifically validated for this subtype are lacking. The GRANT score, incorporating grade, age, nodes, and tumor stage, shows promise but requires extensive validation in pRCC-specific cohorts. This study aimed to evaluate the prognostic value of the GRANT score and develop a novel nomogram for predicting survival in pRCC patients.

**Methods:**

A multi-center retrospective study was conducted. Patients undergoing surgery for pRCC were identified from the SEER database (2004-2015) and formed the training (n=4,001) and internal validation (n=1,689) cohorts. An external validation cohort (n=151) was sourced from a Chinese institution. Overall survival (OS) and cancer-specific survival (CSS) were primary endpoints. The GRANT score was calculated for all patients. Univariate and multivariate Cox analyses identified independent prognostic factors, which were incorporated into nomograms for predicting 1-, 3-, and 5-year OS and CSS. Model performance was assessed using the concordance index (C-index), time-dependent receiver operating characteristic curves, and calibration plots.

**Results:**

Multivariate analysis confirmed the GRANT score as an independent prognostic factor for both OS and CSS. The prognostic nomograms integrated key variables, including surgical approach, marital status, TNM stage, tumor size, Fuhrman grade, and the GRANT score. For OS prediction, the nomogram achieved C-indices of 0.711 (training), 0.720 (internal validation), and 0.740 (external validation). For CSS prediction, the model demonstrated superior performance, with C-indices of 0.860 (training), 0.873 (internal validation), and 0.826 (external validation). Calibration curves showed excellent agreement between predicted and observed outcomes. Risk stratification based on nomogram scores effectively distinguished low-, intermediate-, and high-risk patient groups with significantly different survival.

**Conclusion:**

This study validates the GRANT score as an independent prognostic factor in a large pRCC cohort. The developed and externally validated nomogram provides a clinically useful tool with robust performance, particularly for predicting CSS, facilitating personalized risk assessment and postoperative management for pRCC patients.

## Background

Papillary renal cell carcinoma (pRCC) is the second most common histological subtype of renal cell carcinoma (RCC), accounting for approximately 10%-15% of all RCC cases. This subtype exhibits considerable heterogeneity in its clinical presentation, biological behavior, molecular characteristics, and prognosis (1). The current World Health Organization (WHO) classification system emphasizes that pRCC represents a group of tumors with diverse morphological and molecular spectra, and its subtyping is continually refined to more accurately reflect its biological essence and clinical outcomes (2). For patients with localized and locally advanced RCC, the gold-standard radical treatments include partial and radical nephrectomy. However, clinical follow-up data indicate that postoperative tumor recurrence rates can be as high as 30% (3–5). Notably, a subset of pRCC patients experiences poor overall prognosis, and effective systemic treatment options for advanced pRCC remain limited (6). In this context, establishing reliable prognostic prediction models holds significant clinical value, as it can not only optimize patient counseling and individualized follow-up strategies but also precisely identify high-risk patient populations who may benefit from adjuvant therapy (7).

In recent years, numerous studies have sought to elucidate key prognostic factors for pRCC, including patient performance status (Eastern Cooperative Oncology Group, ECOG score), tumor stage (TNM staging system), maximum tumor diameter, regional lymph node status, nuclear grade (WHO/ISUP grading system), and histopathological features such as necrosis or vascular invasion (8). Based on these parameters, several prognostic prediction models and nomogram tools have been developed. Although these models differ in variable selection, weight assignment, and predictive performance, they all provide valuable references for prognostic assessment in pRCC. Current international guidelines, including those from the European Society for Medical Oncology (ESMO) and the European Association of Urology (EAU), recommend using certain prognostic models to guide postoperative management of pRCC patients; however, they also explicitly highlight the current lack of high-level evidence supporting the preferential selection of any specific model (9). Representative models include: 1) The UISS (University of California Los Angeles Integrated Staging System), developed by Zisman et al. in 2002 and externally validated in a cohort of 4,202 patients in 2012, though its validation cohort did not specifically focus on pRCC patients (10, 11); 2) The 2018 Leibovich model, designed to assess recurrence-free survival (RFS) and cancer-specific survival (CSS) in patients with clear cell RCC (ccRCC), pRCC, and chromophobe RCC, which demonstrated good predictive performance (C-index 0.77–0.83) in a subset of 607 pRCC patients (12); and 3) The VENUSS model, developed by the Klatte team, which focuses on predicting postoperative recurrence risk in non-metastatic pRCC and has been externally validated in a multicenter cohort of 980 non-metastatic pRCC patients (13, 14).

Recently, a GRade, Age, Nodes, and Tumor (GRANT) score was established through an exploratory subgroup analysis of an adjuvant therapy clinical trial based on interleukin-2 and interferon-alpha (15). This scoring system was subsequently independently validated in the ASSURE adjuvant therapy trial population, confirming its significant clinical value in predicting disease-free survival (DFS) and overall survival (OS) (16, 17). The GRANT score integrates four routinely available clinical parameters: Fuhrman nuclear grade, patient age (with a cutoff of 60 years), lymph node status (pathological, pN), and pathological tumor stage (pT stage). Each parameter is assigned a binary score (0 or 1 point), and patients are ultimately stratified into three risk tiers: low-risk (0–1 points), intermediate-risk (2 points), and high-risk (3–4 points).

Although existing prognostic models offer some guidance for the clinical management of pRCC, it is important to note that most were developed from mixed cohorts that included ccRCC. The relatively low incidence of pRCC presents methodological challenges, including insufficient sample sizes, for developing risk stratification tools specifically for this subtype. To address this, our study adopts a multi-center design, integrating data from the Surveillance, Epidemiology, and End Results (SEER) database of the National Cancer Institute and retrospective data from our own institution, to systematically evaluate and optimize the prognostic predictive performance of the GRANT score in pRCC patients. Furthermore, we aim to construct a prognostic nomogram based on multivariate analysis, intending to provide clinicians with a scientifically robust and practical prognostic assessment tool, ultimately facilitating precise and individualized postoperative management for pRCC patients.

## Methods

### Patient selection and study design

This multicenter retrospective study integrated clinical data from the SEER database of the National Cancer Institute and Qingdao University Affiliated Hospital. The SEER database covers approximately 30% of the US population and provides comprehensive data, including clinicopathological characteristics, demographic information, and survival outcomes. Using SEER*Stat software (version 8.4.5), we identified patients diagnosed with pRCC (ICD-O-3 code 8260/3) between 2004 and 2015, resulting in an initial cohort of 15,040 cases. The inclusion criteria were: (1) histopathologically confirmed pRCC; (2) a single primary tumor; and (3) treatment with either partial or radical nephrectomy. Exclusion criteria included: (1) missing data for key variables (age, sex, race, pathological grade, TNM stage, tumor size, tumor laterality, surgical approach, follow-up time, marital status, or survival outcomes); (2) pRCC not being the first primary malignancy; and (3) data sourced solely from autopsy reports or death certificates. Ultimately, 5,690 eligible patients were included. The SEER cohort was randomly split in a 7:3 ratio into a training set (n=4,001) for nomogram development and an internal validation set (n=1,689). This ratio was chosen to ensure model stability while utilizing an independent set to assess generalization capability and prevent overfitting.

**Figure 1:**
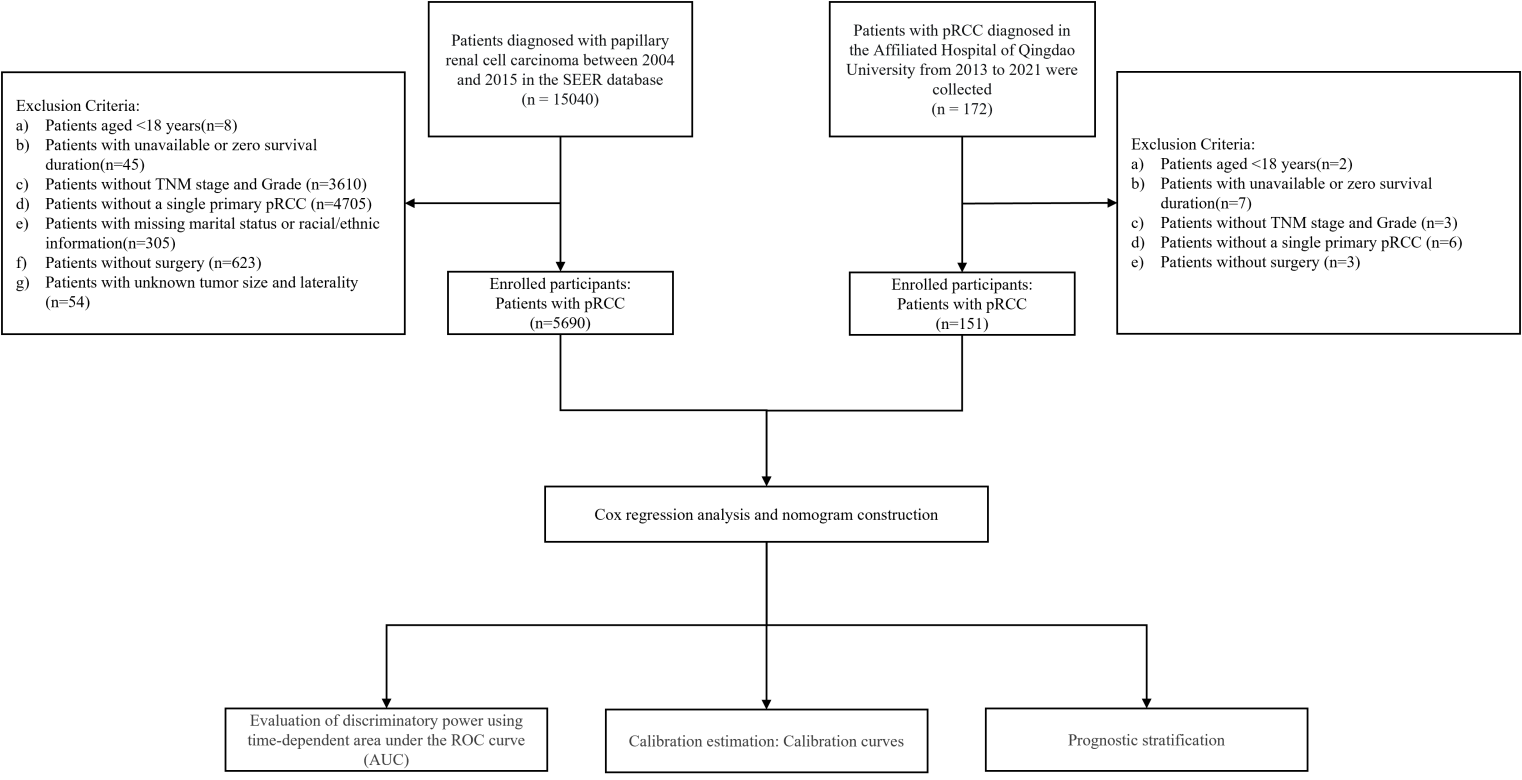
Study flowchart illustrating patient selection process and cohort allocation.

To validate model generalizability, an additional 172 pRCC patients treated at Qingdao University Affiliated Hospital between February 2013 and December 2021 were enrolled as an external validation cohort. The rationale for this multi-center validation strategy was twofold: 1) to leverage the large sample size of the SEER database for robust model construction, and 2) to evaluate the model’s applicability in a real-world clinical setting using an independent external cohort. The inclusion criteria for the external validation set were consistent with those for the training set, with the last follow-up date in December 2024. The study protocol was approved by the Institutional Review Board of Qingdao University Affiliated Hospital. Informed consent was waived due to the retrospective nature of the study. The study flowchart is presented in **Figure 1**.

### Study variables

The following variables were collected: 1) baseline demographic characteristics (age at diagnosis, race, and marital status); 2) tumor characteristics (maximum tumor diameter, histological grade, TNM stage); 3) treatment information (surgical procedure); 4) GRANT score; and 5) survival outcomes (vital status and survival time). The GRANT score was calculated based on four clinicopathological parameters, each assigned a binary score (0 or 1 point) as follows: 1) Tumor grade: Fuhrman nuclear grade 1–2 scored 0 points; grade 3–4 scored 1 point. 2) Age: ≤60 years scored 0 points; >60 years scored 1 point. 3) Primary tumor extent (pT stage): pT1–T3a scored 0 points; pT3b–T4 scored 1 point. 4) Lymph node status: pN0 or pNx (no pathologically confirmed lymph node metastasis) scored 0 points; pN1 (pathologically confirmed lymph node metastasis) scored 1 point. It is noteworthy that for lymph node status assessment, pN0 (pathologically confirmed negative nodes) and pNx (nodes not assessed or status unknown) were combined into a single category. This approach was justified based on: 1) the substantial proportion of pNx cases in the SEER database, thereby enhancing the model’s applicability; 2) consistency with previous studies (17); and 3) recognition of the high false-positive rate associated with radiographic lymph node assessment, whereas this study primarily relied on pathological staging.

TNM staging was performed according to the AJCC 6th edition criteria. The total GRANT score ranged from 0 to 4 points. Patients were stratified into three risk groups for prognostic assessment: low-risk (0–1 points), intermediate-risk (2 points), and high-risk (3–4 points) (see **Supplementary Table 1**). The primary endpoints were OS and CSS. OS was defined as the time from diagnosis to death from any cause, and CSS was defined as the time from diagnosis to death specifically attributed to pRCC.

### Statistical analysis

Continuous variables are presented as mean ± standard deviation or median (interquartile range), while categorical variables are expressed as frequencies (percentages). Kaplan-Meier survival curves were plotted to assess survival differences across GRANT score groups, with inter-group comparisons performed using the log-rank test. A multi-stage variable selection strategy was employed to identify independent prognostic factors. First, univariate Cox regression analysis was conducted for all candidate variables (including age, sex, race, marital status, tumor grade, T stage, N stage, M stage, tumor size, and GRANT score), with statistical significance set at P < 0.05. Variables showing significance in the univariate analysis were subsequently subjected to Least Absolute Shrinkage and Selection Operator (LASSO) regression for dimensionality reduction and feature selection. The optimal penalty parameter (λ) for LASSO was determined via 10-fold cross-validation, retaining predictors with non-zero coefficients. These selected variables were then incorporated into a multivariable Cox proportional hazards regression model using forward LR selection. Finally, a prognostic nomogram was constructed based on the statistically significant variables in the final multivariable model to predict 1-, 3-, and 5-year CSS and OS in pRCC patients.

To evaluate the predictive performance of the GRANT score and the nomogram, both internal and external validations were performed. The concordance index (C-index) and its 95% confidence interval were used to assess the overall discriminative ability of the models. Time-dependent receiver operating characteristic (ROC) curve analysis was used to calculate the area under the curve (AUC) at 1, 3, and 5 years, evaluating the models’ discriminative power at specific time points. Calibration curves were plotted to analyze the agreement between predicted probabilities and observed outcomes. Internal validation of discrimination and calibration was performed using the bootstrap method with 1000 resamples. Furthermore, based on the risk scores derived from the nomogram, optimal cut-off values were determined using X-tile software (version 3.6.1) to categorize patients into low-, intermediate-, and high-risk groups.

All statistical analyses were performed using SPSS 26.0 and R language (version 4.4.3). A two-sided P value < 0.05 was considered statistically significant.

## Results

### Patient baseline characteristics

A total of 5,690 patients with pRCC meeting the inclusion criteria were included in this study, with data sourced from the SEER database. These patients were randomly assigned to a training set (n=4,001) and an internal validation set (n=1,689). Additionally, 151 pRCC patients from our institution were enrolled as an external validation cohort. As shown in **Table 1**, significant differences in baseline characteristics were observed among the cohorts (χ² test, specific P-values are detailed in **Table 1**). In the SEER cohort, male patients accounted for 73.41% (4,177/5,690), and White individuals constituted 69.67% (3,964/5,690). Regarding pathological stage and grade, 51.88% (2,952/5,690) of patients had Fuhrman grade 2, 72.58% (4,130/5,690) were classified as T1 stage (AJCC 6th edition), and 76.19% (4,335/5,690) were in GRANT grade 1. In the external validation cohort, male patients accounted for 70.20% (106/151), 43.71% (66/151) had Fuhrman grade 2, 38.41% (58/151) were T1 stage, and 71.52% (108/151) were in GRANT grade 1. Other detailed clinicopathological characteristics are presented in **Table 1**.

**Table 1:** Demographic and clinicopathological characteristics of patients in the training and validation cohorts.

Characteristic	Training cohort	Internal validation cohort	External validation cohort	P value
	(n = 4001), n (%)	(n = 1689), n (%)	(n = 151), n (%)	
Age				< 0.001
≤60	2013(50.31%)	869(51.45%)	50(33.11%)	
>60	1988(49.69%)	820(48.55%)	101(66.89%)	
Sex				0.63
Female	1058(26.44%)	455(26.94%)	45(29.80%)	
Male	2943(73.56%)	1234(73.06%)	106(70.20%)	
Race				< 0.001
White	2789(69.71%)	1175(69.57%)	0	
Black	1046(26.14%)	433(25.64%)	0	
Others	166(4.15%)	81(4.79%)	151	
Marital status				< 0.001
Married	2533(63.31%)	1054(62.40%)	121(80.13%)	
Others	1468(36.69%)	635(37.60%)	30(19.87%)	
Grade				< 0.01
1	445(11.12%)	183(10.84%)	31(20.53%)	
2	2083(52.06%)	869(51.45%)	66(43.71%)	
3	1332(33.29%)	565(33.45%)	44(29.14%)	
4	141(3.52%)	72(4.26%)	10(6.62%)	
T stage				< 0.001
T1	2881(72.00%)	1249(73.95%)	58(38.41%)	
T2	532(13.30%)	204(12.08%)	10(6.62%)	
T3	563(14.07%)	231(13.68%)	78(51.66%)	
T4	25(0.63%)	5(0.29%)	5(3.31%)	
N stage				0.08
N0	3774(94.33%)	1595(94.44%)	136(90.07%)	
N1	227(5.67%)	94(5.56%)	15(9.93%)	
M stage				0.43
M0	3861(96.50%)	1623(96.09%)	148(98.01%)	
M1	140(3.50%)	66(3.91%)	3(1.99%)	
Tumor size	5.10 ± 3.99	5.09 ± 5.32	4.25 ± 2.80	
Surgical approach				< 0.001
Partial	1676(41.90%)	752(44.50%)	85(56.30%)	
Radical	2325(58.10%)	937(55.50%)	66(43.70%)	
GRANT				0.20
1	3043(76.06%)	1292(76.49%)	108(71.52%)	
2	784(19.60%)	337(19.96%)	32(21.19%)	
3	174(4.34%)	60(3.55%)	11(7.29%)	

### Survival analysis based on GRANT score and prognostic factor assessment

**Figure 2:**
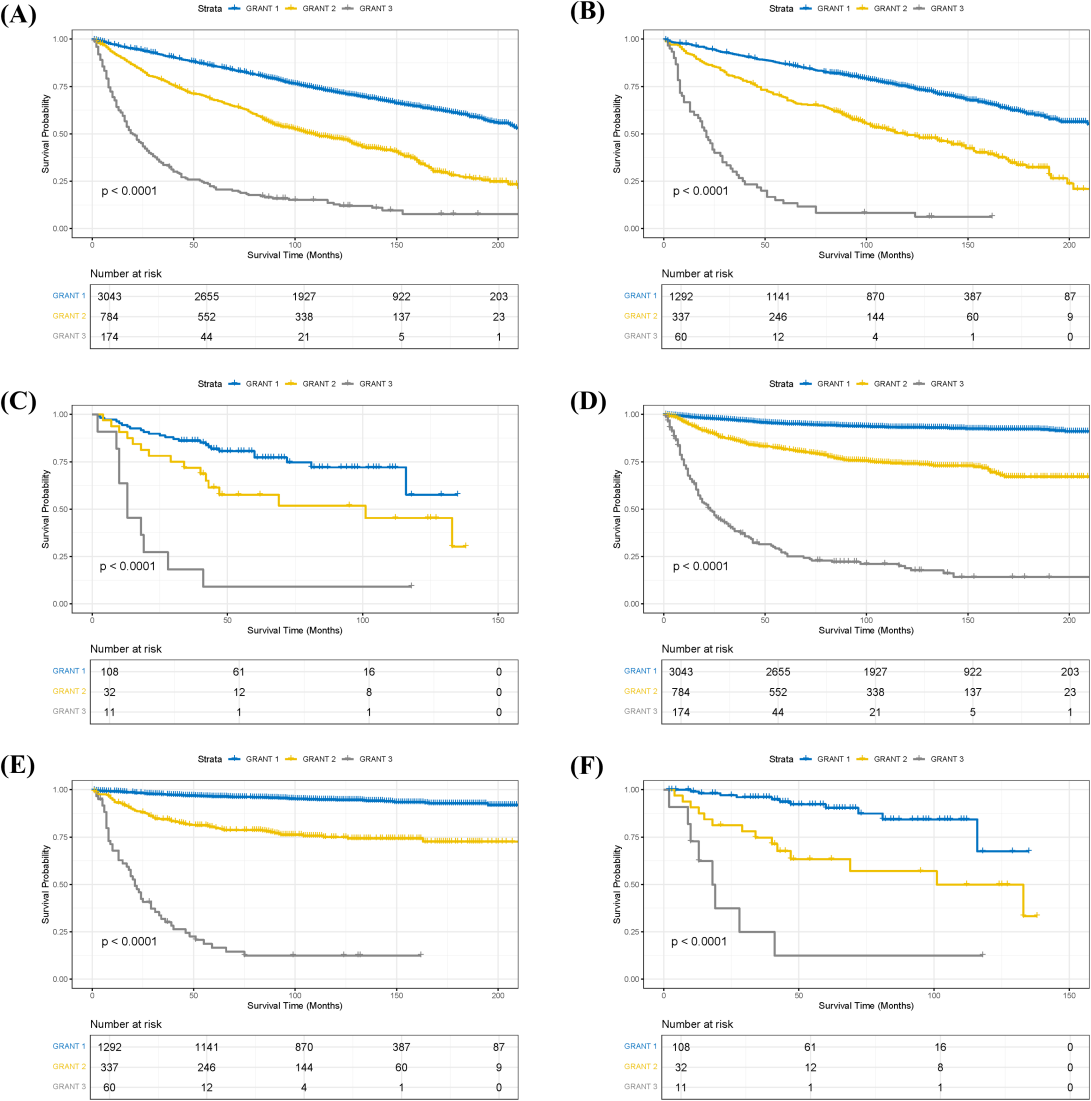
Kaplan-Meier survival curves stratified by GRANT score. **(A)** Overall survival (OS) in the training cohort. **(B)** OS in the internal validation cohort. **(C)** OS in the external validation cohort. **(D)** Cancer-specific survival (CSS) in the training cohort. **(E)** CSS in the internal validation cohort. **(F)** CSS in the external validation cohort.

Stratified analysis of OS and CSS was performed for pRCC patients according to their GRANT scores. Kaplan-Meier survival curves demonstrated significant differences in both OS and CSS among the different GRANT score groups in both the training and validation cohorts (log-rank test, all P < 0.05; **Figure 2**). To further evaluate the predictive performance of the GRANT score, the C-index and time-dependent AUC at 1, 3, and 5 years were calculated in the training cohort. For OS, the C-index was 0.621 in the training cohort, with 1-, 3-, and 5-year AUCs of 0.696, 0.675, and 0.657, respectively. In the internal validation cohort, the C-index for OS was 0.621, with corresponding AUCs of 0.697, 0.671, and 0.662. The external validation cohort showed a C-index of 0.661 for OS, with AUCs of 0.676, 0.696, and 0.675, respectively. The corresponding results for CSS are detailed in **Supplementary Table 2**.

To identify independent prognostic factors for OS and CSS, univariate Cox regression, followed by LASSO regression, and finally, multivariable Cox proportional hazards regression with forward stepwise selection were performed sequentially in the training set. Univariate analysis revealed that marital status, TNM stage (AJCC 6th edition), maximum tumor diameter, Fuhrman grade, and GRANT score were significantly associated with OS (all P < 0.05). TNM stage, maximum tumor diameter, Fuhrman grade, and GRANT score were significantly associated with CSS (all P < 0.05). Subsequently, variables with statistical significance from the univariate analysis were included in the LASSO regression for further screening (**Supplementary Figure 1**), and the final retained variables were incorporated into the multivariable Cox regression model.

Multivariable analysis identified seven independent prognostic factors for OS, including marital status, TNM stage, maximum tumor diameter, Fuhrman grade, and GRANT score (**Table 2**). Six factors were significantly associated with CSS, namely TNM stage, maximum tumor diameter, Fuhrman grade, and GRANT score (**Table 3**). Notably, the widely used TNM staging system was confirmed as an independent prognostic factor for both OS and CSS in this study, consistent with previous findings. Furthermore, multivariable analysis validated the GRANT score as an independent predictor for both OS and CSS. Additionally, marital status was identified as a potential protective factor for OS.

**Table 2:** Univariate and forward stepwise multivariate Cox proportional hazards regression analyses of OS in the training cohort.

Variables	Univariate analysis			Multivariate analysis		
	HR	95% CI	P value	HR	95% CI	P value
Sex						
Female	Ref					
Male	1.034	0.925-1.157	0.552			
Race						
Others	Ref					
Black	0.988	0.759-1.286	0.930			
White	0.996	0.774-1.281	0.974			
Marital status						
Married	Ref			Ref		
Others	1.302	1.179-1.439	<0.001	1.347	1.218-1.489	<0.001
Grade						
1	Ref			Ref		
2	1.286	1.070-1.545	<0.01	1.211	1.007-1.457	0.041
3	1.988	1.649-2.396	<0.001	0.906	0.726-1.131	0.385
4	4.798	3.704-6.216	<0.001	1.330	0.988-1.790	0.060
T stage						
T1	Ref					
T2	1.739	1.516-1.994	<0.001			
T3	2.771	2.454-3.130	<0.001			
T4	7.789	5.049-12.014	<0.001			
N stage						
N0	Ref			Ref		
N1	7.238	6.236-8.400	<0.001	1.819	1.458-2.268	<0.001
M stage						
M0	Ref			Ref		
M1	9.368	7.798-11.256	<0.001	3.627	2.914-4.514	<0.001
Tumor size	1.056	1.050-1.062	<0.001	1.015	1.006-1.025	0.002
Surgical approach						
Partial	Ref			Ref		
Radical	2.582	2.305-2.892	<0.001	1.992	1.767-2.244	<0.001
GRANT						
1	Ref			Ref		
2	2.396	2.145-2.676	<0.001	2.434	2.057-2.881	<0.001
3	8.021	6.745-9.539	<0.001	3.326	2.520-4.391	<0.001

**Table 3:** Univariate and forward stepwise multivariate Cox proportional hazards regression analyses of CSS in the training cohort.

Variables	Univariate analysis			Multivariate analysis		
	HR	95% CI	P value	HR	95% CI	P value
Sex						
Female	Ref					
Male	1.030	0.847-1.252	0.770			
Race						
Others	Ref					
Black	0.644	0.416-0.996	0.048			
White	0.900	0.601-1.348	0.609			
Marital status						
Married	Ref					
Others	1.042	0.871-1.246	0.654			
Grade						
1	Ref			Ref		
2	1.910	1.172-3.113	<0.01	1.549	0.949-2.530	0.080
3	6.047	3.754-9.742	<0.001	1.960	1.164-3.300	0.011
4	21.322	12.693-35.818	<0.001	3.101	1.749-5.498	<0.001
T stage						
T1	Ref			Ref		
T2	4.855	3.810-6.185	<0.001	2.413	1.851-3.144	<0.001
T3	10.978	8.939-13.482	<0.001	2.692	2.057-3.523	<0.001
T4	32.844	19.842-54.366	<0.001	4.410	2.527-7.694	<0.001
N stage						
N0	Ref			Ref		
N1	19.610	16.252-23.661	<0.001	2.614	1.965-3.476	<0.001
M stage						
M0	Ref			Ref		
M1	21.459	17.271-26.663	<0.001	3.315	2.543-4.323	<0.001
Tumor size	1.069	1.063-1.075	<0.001	1.024	1.012-1.035	<0.001
Surgical approach						
Partial	Ref			Ref		
Radical	6.633	5.048-8.716	<0.001	2.670	1.986-3.591	<0.001
GRANT						
1	Ref			Ref		
2	4.332	3.550-5.286	<0.001	1.908	1.428-2.550	<0.001
3	25.814	20.601-32.345	<0.001	2.490	1.666-3.721	<0.001

### Construction and application of the prognostic nomogram

**Figure 3:**
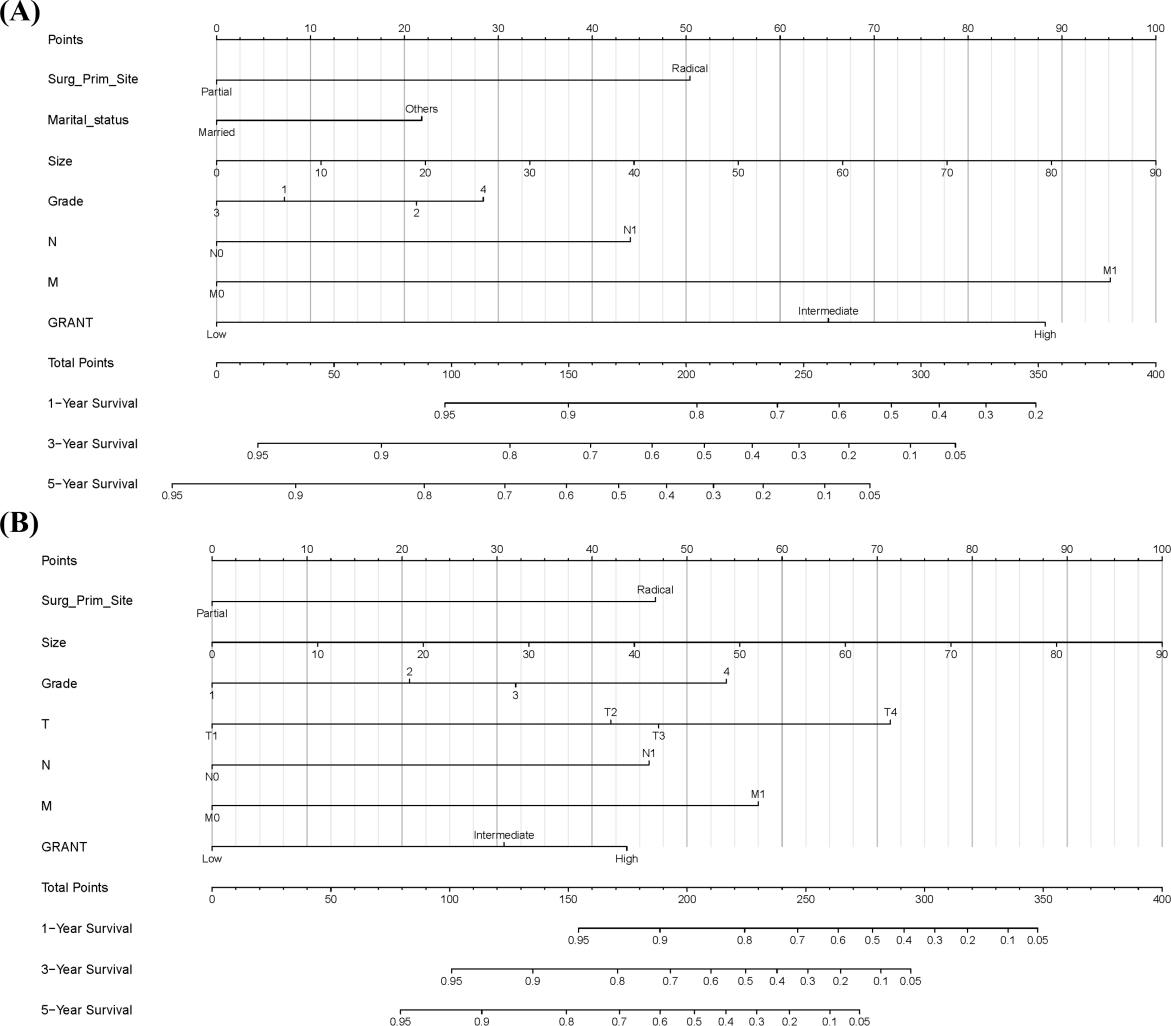
Prognostic Nomograms. **(A)** Prognostic nomogram for predicting 1-year, 3-year, and 5-year overall survival (OS). **(B)** Prognostic nomogram for predicting 1-year, 3-year, and 5-year cancer-specific survival (CSS).

Based on the results of the multivariable Cox regression analysis, we constructed nomograms to predict prognosis in pRCC patients. The analysis indicated that surgical approach at diagnosis, marital status, N stage, M stage, maximum tumor diameter, Fuhrman grade, and GRANT score were independent prognostic factors for 1-, 3-, and 5-year OS (**Figure 3A**). Concurrently, surgical approach, TNM stage, maximum tumor diameter, Fuhrman grade, and GRANT score were confirmed as independent prognostic factors for 1-, 3-, and 5-year CSS (**Figure 3B**). The nomogram is used as follows: first, for each predictor variable, locate its value on the top scale and draw a vertical line downward to the Points axis to determine the assigned score. Second, sum the scores obtained for all variables to get the Total Points. Finally, locate the total score on the respective bottom scales for 1-, 3-, and 5-year survival probability to obtain the predicted survival probability for the patient.

### Validation and evaluation of the predictive model

Time-dependent ROC curve analysis was employed to systematically evaluate the predictive performance of the nomogram for survival rates calculated on a monthly basis. The C-index and the AUC were used as quantitative indicators of the model’s discriminative ability. These metrics theoretically range from 0.5 (no discrimination) to 1.0 (perfect discrimination), with higher values indicating superior discriminative power.

**Figure 4:**
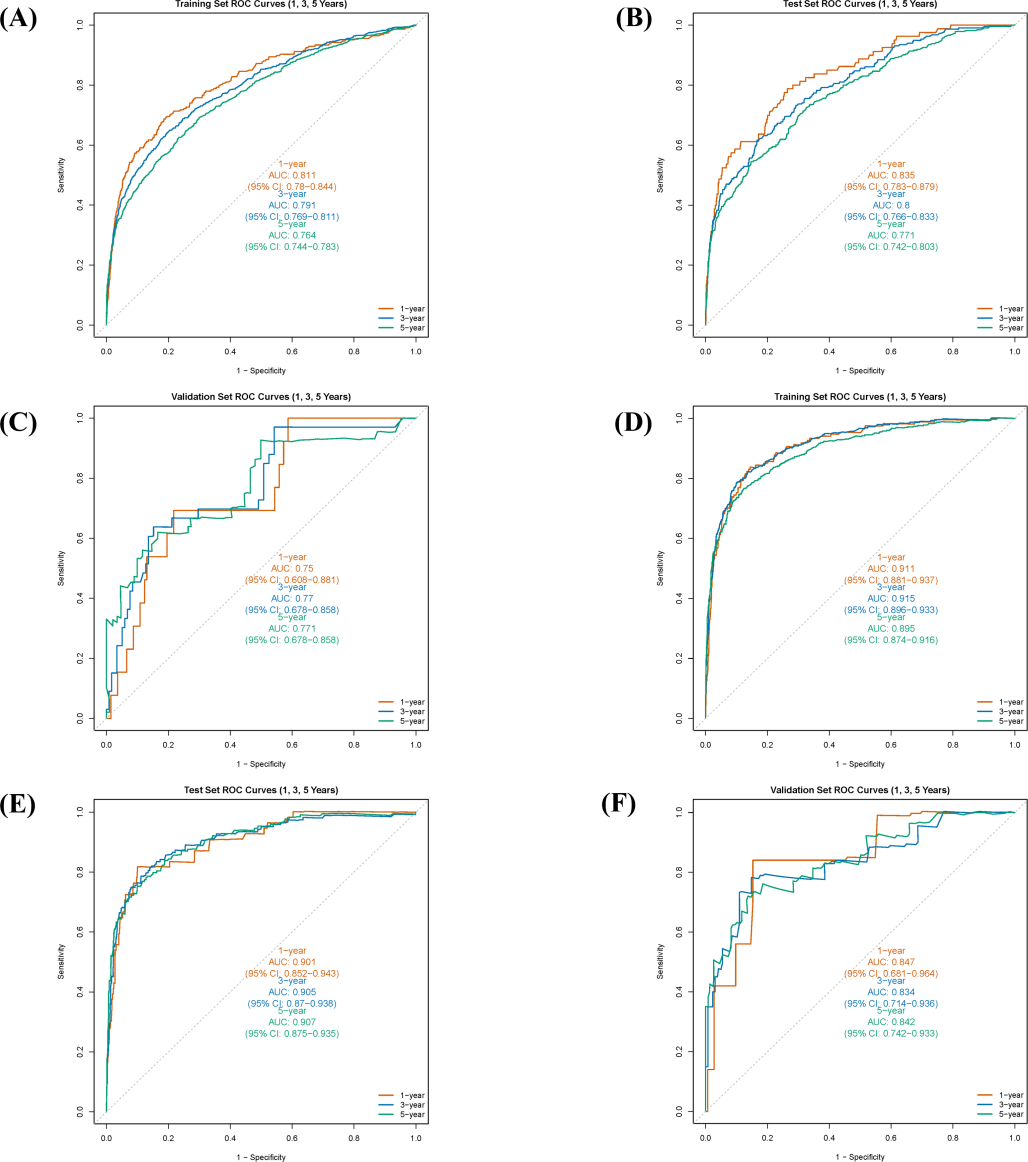
Receiver operating characteristic (ROC) curves and corresponding area under the curve (AUC) values for the nomogram in predicting 1-, 3-, and 5-year overall survival (OS) and cancer-specific survival (CSS) in different cohorts. **(A)** ROC curves for OS in the training cohort. **(B)** ROC curves for OS in the internal validation cohort. **(C)** ROC curves for OS in the external validation cohort. **(D)** ROC curves for CSS in the training cohort. **(E)** ROC curves for CSS in the internal validation cohort. **(F)** ROC curves for CSS in the external validation cohort.

The results demonstrated that the nomogram model exhibited good predictive accuracy across the training, internal validation, and external validation sets. For OS prediction, the C-index was 0.711 (95% CI: 0.697-0.724) in the training set, with 1-, 3-, and 5-year AUC values of 0.811 (95% CI: 0.780-0.844), 0.791 (95% CI: 0.769-0.811), and 0.764 (95% CI: 0.744-0.783), respectively (**Figure 4A**). In the internal validation set, the C-index was 0.720 (95% CI: 0.700-0.741), with corresponding AUCs of 0.835 (95% CI: 0.783-0.879), 0.800 (95% CI: 0.766-0.833), and 0.771 (95% CI: 0.742-0.803) (**Figure 4B**). The external validation set yielded a C-index of 0.740 (95% CI: 0.665-0.814) for OS, with AUCs of 0.750 (95% CI: 0.608-0.881), 0.770 (95% CI: 0.678-0.858), and 0.771 (95% CI: 0.678-0.858) (**Figure 4C**).

For CSS prediction, the C-index was 0.860 (95% CI: 0.843-0.876) in the training set, with 1-, 3-, and 5-year AUC values of 0.911 (95% CI: 0.881-0.937), 0.915 (95% CI: 0.896-0.933), and 0.895 (95% CI: 0.874-0.916), respectively (**Figure 4D**). In the internal validation set, the C-index was 0.873 (95% CI: 0.849-0.898), with AUCs of 0.901 (95% CI: 0.852-0.943), 0.905 (95% CI: 0.870-0.938), and 0.907 (95% CI: 0.875-0.935) (**Figure 4E**). The external validation set showed a C-index of 0.826 (95% CI: 0.733-0.900) for CSS, with AUCs of 0.847 (95% CI: 0.681-0.964), 0.843 (95% CI: 0.714-0.936), and 0.842 (95% CI: 0.742-0.933) (**Figure 4F**). These results indicate that the predictive model developed in this study possesses high discriminative accuracy across different datasets.

**Figure 5:**
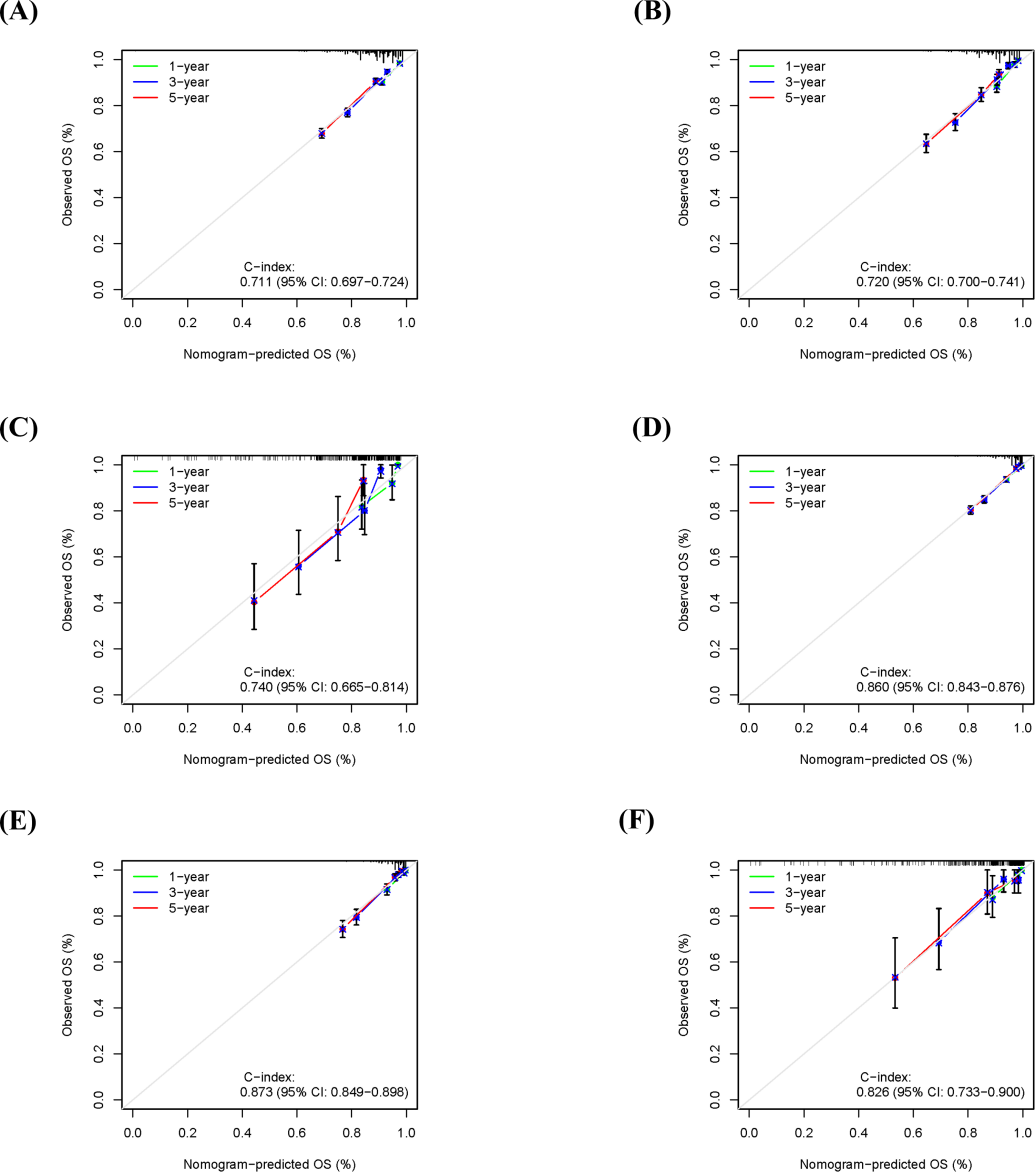
Calibration curves for the nomogram in predicting 1-, 3-, and 5-year overall survival (OS) and cancer-specific survival (CSS) in different cohorts. **(A)** OS calibration curve for the training cohort. **(B)** OS calibration curve for the internal validation cohort. **(C)** OS calibration curve for the external validation cohort. **(D)** CSS calibration curve for the training cohort. **(E)** CSS calibration curve for the internal validation cohort. **(F)** CSS calibration curve for the external validation cohort.

Furthermore, calibration curve analysis was used to assess the accuracy of the predicted 1-, 3-, and 5-year survival probabilities in the training, internal validation, and external validation sets. Under ideal calibration, predicted values should align perfectly with the 45-degree reference line. As shown in **Figures 5A–C**, for OS prediction, the calibration curves demonstrated good agreement between the nomogram’s predictions and the actual observations, confirming satisfactory calibration capability. Similarly, the calibration curves for CSS prediction also showed good consistency (**Figures 5D–F**), further validating the model’s calibration performance.

### Evaluation of the nomogram’s risk stratification ability

**Figure 6:**
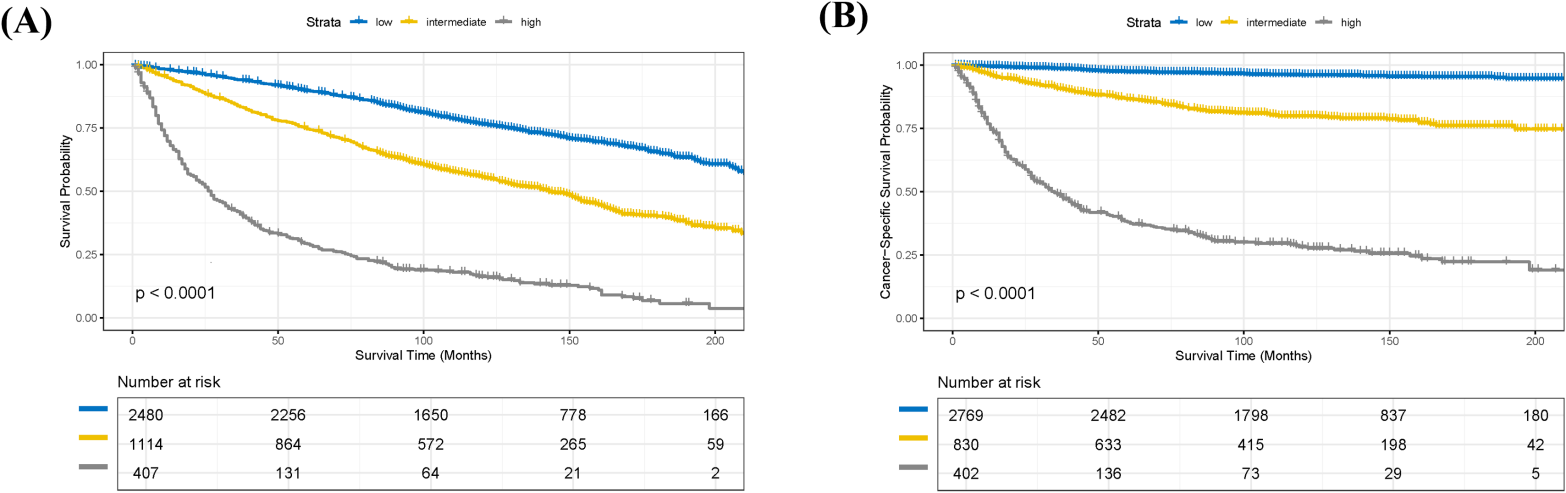
Kaplan-Meier survival analysis of patients in the training cohort stratified by risk groups. **(A)** Kaplan-Meier curves for overall survival (OS) demonstrating significant differences among low-, intermediate-, and high-risk groups (p < 0.0001). **(B)** Kaplan-Meier curves for cancer-specific survival (CSS) demonstrating significant differences among low-, intermediate-, and high-risk groups (p < 0.0001).

Based on the nomogram model, we calculated a risk score for each patient in the training cohort and performed risk stratification accordingly. Using X-tile software (version 3.6.1), optimal cut-off values were determined to categorize patients into three subgroups: low-risk, intermediate-risk, and high-risk (18). For the OS nomogram, the stratification criteria were: low-risk (score ≤ 2.51), intermediate-risk (2.51 < score ≤ 6.04), and high-risk (score > 6.04) (**Supplementary Figures 2A, B**). For the CSS nomogram, the criteria were: low-risk (score ≤ 7.34), intermediate-risk (7.34 < score ≤ 27.23), and high-risk (score > 27.23) (**Supplementary Figures 2C, D**). Kaplan-Meier survival analysis revealed statistically significant differences in OS among the different risk subgroups in the training cohort (**Figure 6A**). Similarly, the risk stratification for CSS also showed statistically significant differences (**Figure 6B**).

## Discussion

RCC is the most common malignant kidney tumor in adults, accounting for approximately 90% of all kidney cancers, and its incidence continues to rise globally (19). In the United States, RCC constituted about 4.1% of new cancer cases in 2022 (20). Papillary RCC (pRCC) is the most frequent subtype among non-clear cell RCCs. Although its overall incidence is relatively low (21), pRCC exhibits distinct differences from ccRCC in terms of clinical presentation, molecular features, disease prognosis, and treatment response (22). This biological heterogeneity forms the rationale for developing pRCC-specific prognostic tools. Current international guidelines (e.g., ESMO/EAU) recommend using prognostic models to guide postoperative adjuvant therapy decisions; however, existing models (e.g., UISS, Leibovich) were predominantly developed from mixed cohorts including ccRCC, leaving a significant evidence gap for prognostic tools specifically designed and extensively validated for pRCC. To address this, our study employed a multi-center design, integrating the US SEER database and our institutional data, to systematically evaluate the prognostic value of the GRANT score in pRCC patients and to explore the construction of a predictive model optimized for the biological characteristics of pRCC.

This study confirmed that the GRANT score is an independent predictor for both OS and CSS in pRCC patients (multivariable Cox regression, all P < 0.05). In the training set, the GRANT score demonstrated good discriminatory ability, with a C-index of 0.621 for OS and 0.732 for CSS. The nomogram prediction model constructed based on these findings showed robust predictive performance in both the training and external validation sets, exhibiting excellent performance especially for CSS prediction (1-, 3-, and 5-year AUCs all > 0.80 in the training set). Calibration curve analysis indicated high consistency between predicted probabilities and actual survival rates. Furthermore, risk stratification based on the nomogram successfully identified subgroups with significantly different survival outcomes (comparisons among low-, intermediate-, and high-risk groups for both OS and CSS, all P < 0.001).

For surgically treated pRCC patients, various prognostic tools developed in recent years, integrating clinical and pathological features, allow for more accurate assessment of recurrence risk (8, 23). However, although increasing the number of model variables might enhance predictive accuracy, the associated complexity could limit their application in routine clinical practice (24). Therefore, balancing predictive efficacy with operational simplicity is crucial for facilitating the clinical adoption of such models (23, 24). Among the models mentioned in international pRCC guidelines, the GRANT score has been recognized as a simple tool for predicting prognosis after pRCC resection (24). Its clinical applicability was confirmed in a large nationwide study by Juul et al. (25); Maffezzoli et al. further validated the reliability of this score through a three-risk-group stratification analysis of pRCC patients undergoing resection using real-world data (26). Additionally, a recent evaluation by Piccinelli et al. in a North American population assessed the VENUSS and GRANT models for predicting 5-year cancer-specific survival after surgery in non-metastatic pRCC patients. Their results showed that the GRANT risk classification achieved an accuracy of 0.65 in cross-validation, which, while superior to random prediction in decision curve analysis, performed less well than the VENUSS model (C-index: 0.73) (27).

Our findings provide important complementary evidence to the existing literature: First, the GRANT score was originally derived from an adjuvant therapy clinical trial (subgroup analysis of the ASSURE trial) (17), whereas our study is the first to validate its independent prognostic value in a large pRCC cohort, supporting its generalizability as a concise clinical tool. Second, our results demonstrate that the pRCC-specific nomogram we developed exhibits excellent performance in CSS prediction (3-year CSS AUC: 0.845 in the external validation set), which is comparable to the performance of previously validated pRCC-specific models, such as VENUSS for CSS prediction (28–30). It is important to note that, as our study did not directly compare the performance of models like UISS and Leibovich with our model on the same dataset, we refrain from claiming “superiority.” Nonetheless, our results confirm that a model specifically constructed for pRCC, integrating the GRANT score and other readily available clinical parameters, can achieve predictive accuracy comparable to existing well-established models, offering a validated new option for precise prognostic assessment in pRCC.

The parameters included in the GRANT score, such as age and tumor grade, align well with core prognostic factors for pRCC, while the incorporation of lymph node status further refines the accuracy of metastasis risk assessment. Furthermore, our study identified marital status as an independent predictor for OS. This likely reflects the combined influence of non-biological factors—such as social support, socioeconomic status, and adherence to medical care—on overall survival, rather than a direct biological effect. This finding suggests that sociodemographic factors warrant clinical attention when evaluating a patient’s overall prognosis.

The primary strength of this study lies in its multi-center validation integrating the US SEER database (n = 5,690) and our institutional cohort (n = 151), which significantly enhanced statistical power and improved the external validity of the conclusions. The developed nomogram effectively combines the GRANT score with routine clinical parameters, providing clinicians with an intuitive prognostic assessment tool. However, this study has several limitations. First, as a retrospective study, it is inherently susceptible to selection bias. Second, the lack of a head-to-head comparison of our model with existing models like UISS and Leibovich on the same dataset limits definitive conclusions regarding their relative performance. Future studies should conduct such direct comparisons to clarify the strengths and weaknesses of different models in pRCC. Third, due to data availability constraints, we could not comprehensively collect information on certain key prognostic factors, particularly detailed data on socioeconomic status and systemic therapy. Finally, the sample size of our external validation cohort was relatively limited, and the conclusions require further confirmation through larger, independent cohorts.

## Conclusion

Notwithstanding the aforementioned limitations, this study validates the independent prognostic value of the GRANT score in a large pRCC cohort and successfully establishes a nomogram model with good discriminative ability and calibration. This tool demonstrated excellent performance, particularly in predicting CSS, providing clinicians with a practical and specialized reference for assessing postoperative risk in pRCC patients. Its potential clinical utility lies in its ability to aid in the identification of truly high-risk patients, thereby potentially optimizing adjuvant therapy decisions and the formulation of follow-up strategies. However, the realization of this potential requires further validation in prospective studies.

## Data Availability

The original contributions presented in the study are included in the article/**Supplementary Material**. Further inquiries can be directed to the corresponding author.
