# Supplementary material for: Prognostic value of the GRANT score and development of a nomogram in papillary renal cell carcinoma: a SEER-based study with external validation in a Chinese cohort
Source: Front Oncol. 2025 Nov 5;15:1659055. doi: 10.3389/fonc.2025.1659055 (PMC12626819; doi:10.3389/fonc.2025.1659055)
Supplement: Supplementary file 1 [file DataSheet1.docx]

*
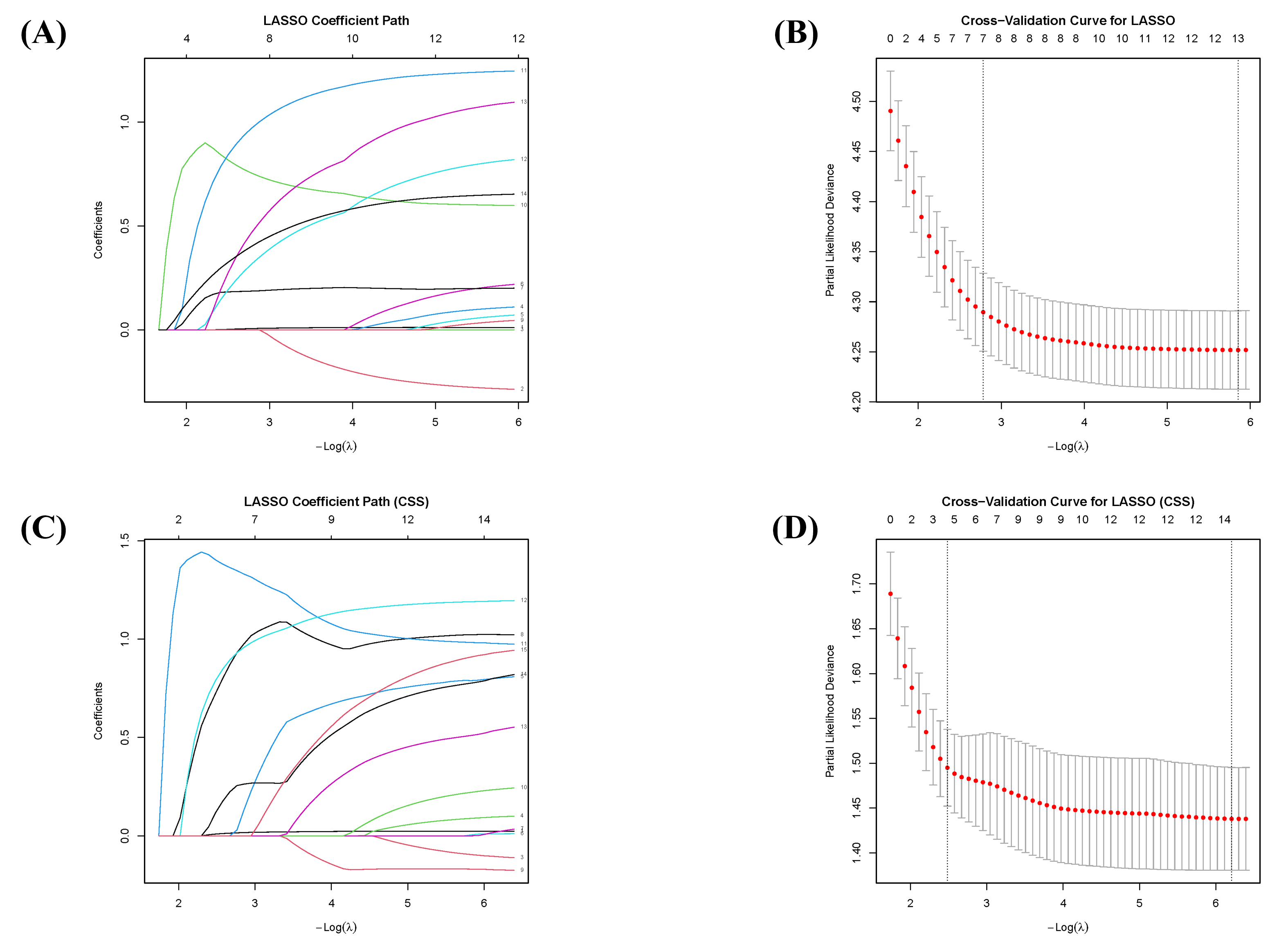
*

*Supplementary Figure 1. Least Absolute Shrinkage and Selection Operator (LASSO) regression analysis for predictor selection of overall survival (OS) and cancer-specific survival (CSS) in patients with papillary renal cell carcinoma. (A) LASSO coefficient profile plot for OS. The vertical dashed line indicates the value selected by 10-fold cross-validation via minimum criteria. (B) Cross-validation curve for OS. The area between the dotted vertical lines represents the optimal lambda (λ) value, where the model achieves the best performance with the fewest predictors. (C) LASSO coefficient profile plot for CSS. (D) Cross-validation curve for CSS.*

*
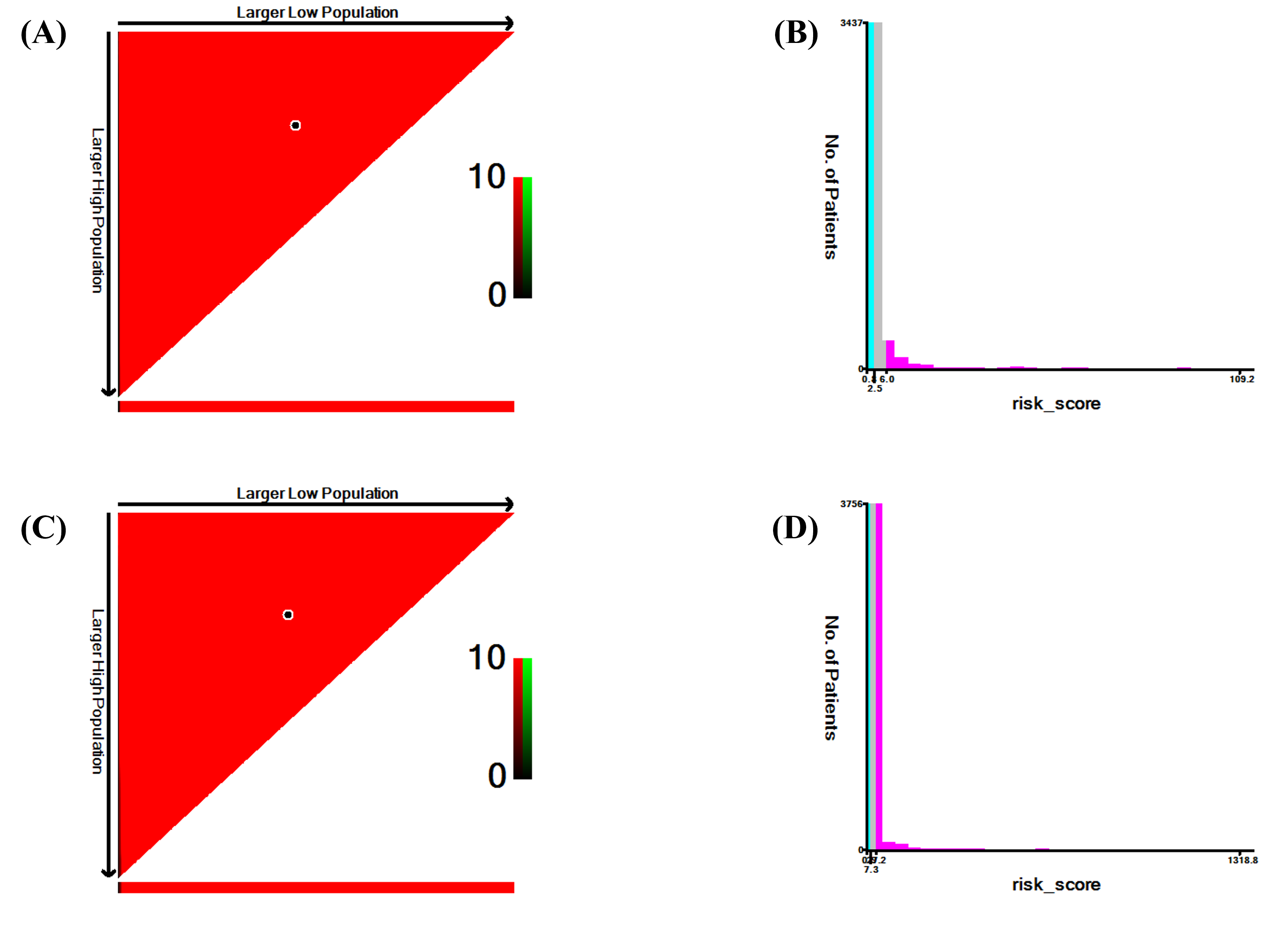
*

*Supplementary Figure 2. Risk stratification of patients in the training cohort based on nomogram-derived risk scores using X-tile analysis. (A) X-tile plot indicating the optimal cut-off points for overall survival (OS) risk score stratification. (B) Histogram illustrating the distribution of OS risk scores among patients, with demarcation of low-, intermediate-, and high-risk groups. (C) X-tile plot indicating the optimal cut-off points for cancer-specific survival (CSS) risk score stratification. (D) Histogram illustrating the distribution of CSS risk scores among patients, with demarcation of low-, intermediate-, and high-risk groups.*
