# Supplementary material for: Prognostic value of the GRANT score and development of a nomogram in papillary renal cell carcinoma: a SEER-based study with external validation in a Chinese cohort
Source: Front Oncol. 2025 Nov 5;15:1659055. doi: 10.3389/fonc.2025.1659055 (PMC12626819; doi:10.3389/fonc.2025.1659055)
Supplement: Supplementary file 2 [file Table1.docx]

Supplementary Table 1. GRANT scoring system: Criteria and risk stratification for prognostic assessment in patients with papillary renal cell carcinoma.

| Variable | Score |
| --- | --- |
| Age |  |
| ＞60 | 1 |
| ≤60 | 0 |
| pT (TNM 2002^a^) |  |
| T1/T2/T3a | 0 |
| T3b/T3c/T4 | 1 |
| Pathologic nodal status |  |
| N0/NX | 0 |
| N1 | 1 |
| Fuhrman grade |  |
| 1/2 | 0 |
| 3/4 | 1 |
| Risk groups |  |
| Low risk | 0/1 |
| Intermediate risk | 2 |
| High risk | 3/4 |
| ^a^TNM according to 2002 TNM Staging (American Joint Committee on Cancer 6th edition). | |

Supplementary Table 2. Discriminatory ability of the GRANT prognostic model for overall survival (OS) and cancer-specific survival (CSS) in patients with papillary renal cell carcinoma.

| Endpoint |  | AUC | | |
| --- | --- | --- | --- | --- |
|  | C-index | 1-Year | 3-Year | 5-Year |
| OS |  |  |  |  |
| Training | 0.621 | 0.696 | 0.675 | 0.657 |
| Internal Validation | 0.621 | 0.697 | 0.671 | 0.662 |
| External Validation | 0.661 | 0.676 | 0.696 | 0.675 |
| CSS |  |  |  |  |
| Training | 0.732 | 0.785 | 0.773 | 0.753 |
| Internal Validation | 0.759 | 0.809 | 0.797 | 0.791 |
| External Validation | 0.764 | 0.832 | 0.824 | 0.768 |
| OS, overall survival; CSS, cancer-specific survival; C-index, concordance index; AUC, area under the time-dependent receiver operating characteristic curve. | | | | |
